# The Role of Probiotics in Preventing Allergic Disease

**DOI:** 10.3390/children6020024

**Published:** 2019-02-05

**Authors:** Helen T. Wang, Sara Anvari, Katherine Anagnostou

**Affiliations:** 1 Department of Pediatrics, Section of Immunology, Allergy, Rheumatology, and Retrovirology, Texas Children’s Hospital, Houston, TX 77030, USA; Helen.Wang@bcm.edu (H.T.W.); Sara.Anvari@bcm.edu (S.A.); 2 Baylor College of Medicine, Department of Pediatrics, Section of Immunology, Allergy, Rheumatology, and Retrovirology, Houston, TX 77030, USA

**Keywords:** probiotics, microbiome, dysbiosis, atopy, food allergy, eczema, allergic rhinitis, asthma

## Abstract

The prevalence of allergic disorders has been increasing worldwide and significantly impacts the quality of life of the atopic individual. There has been an increased interest in the role of probiotics for the prevention and treatment of allergic disorders, given the recent evidence that atopy risk may be associated with a dysbiosis of the gut microbiome. Research in this area is ongoing with some studies showing possible benefits of probiotics, with seemingly little to no risk. While these studies suggest that there may be a promise in probiotic use for the prevention or treatment of allergy, further evidence is needed to determine its efficacy, optimal dosing, and strains needed for treatment. In this review, we discuss recently published studies examining the benefits, risks, and role of probiotics in preventing atopic dermatitis, asthma, allergic rhinitis, and food allergy.

## 1. Introduction

Allergic diseases are increasing in prevalence worldwide and pose a significant burden on society, both economically and psychosocially [1]. Despite their ubiquity, the etiology of allergic disorders remains unknown. In 1989, it was hypothesized that decreased exposure to microbes resulted in an immune system imbalance, favoring a shift toward an allergic response. This hypothesis was based on an observation of decreased incidence of hay fever and eczema in children living with older siblings within large families (resulting in increased microbial exposure) [2]. In more recent times, it has been demonstrated that dysbiosis of the gut microbiome can be associated with an increased risk of atopy [3]. Increasingly, probiotics (the “good bacteria”) have been used in an attempt to correct this.

Probiotics are defined as “live microorganisms which confer a beneficial effect on the host” according to the World Health Organization (WHO) [4]. It has been suggested that probiotics may prevent the allergic response due to their anti-inflammatory effects, although this area remains controversial [5]. There are multiple mechanisms that have been proposed, via which, probiotics decrease atopy, including skewing of the Th1/Th2 balance toward Th1 by inhibiting Th2 cytokines or indirectly increasing IL-10 and T-regulatory cell production through either dendritic cell maturation or toll-like receptors, although the exact pathway remains to be elucidated [6].

We present a review of recently published studies examining the role of probiotics in preventing atopic dermatitis, asthma, allergic rhinitis, and food allergy.

## 2. Atopic Dermatitis

Atopic dermatitis is the most common chronic inflammatory skin disease and is often the first step in the atopic march [1]. The two primary theories on the origins of atopic dermatitis are the “inside-out” concept, which speculates that imbalances in the enteric microbiota result in inflammatory processes and the “outside-in” hypothesis, which suggests that disrupted skin microbiome is the primary triggering event for atopic dermatitis [7]. The idea of probiotic supplementation to restore balance in the microbiome of humans is the foundational argument for the use of probiotics in primary prevention of atopic dermatitis. Three systematic reviews have so far investigated probiotic supplementation in terms of eczema prevention.

The first systematic review by Zuccotti et al. [8] included randomized-controlled trials that examined probiotic administration to mothers starting in pregnancy and/or in the first week of infant life, alongside probiotic administration to high-risk infants and toddlers. Those who had one or more family members with eczema, asthma, gastrointestinal allergy, allergic urticaria, or allergic rhino-conjunctivitis were considered high-risk. Studies that had an age of supplementation greater than 3 months old that included prebiotics, synbiotics, or probiotics in formula milk were considered, and non-human or non-clinical trials were excluded. In this review, 17 studies met the inclusion criteria. Data from 4755 children were analyzed with 2381 in the probiotic group and 2374 in the control group. All studies, except for two, started probiotics during pregnancy (the other two administered probiotics to infants within 48 h of delivery). It was shown that fewer children in the probiotic group developed eczema (28.22% versus 35.67%; RR 0.78; 95% CI: 0.69–0.89; *p* = 0.0003) with the number of children needed to be treated being 13. However, the strains of probiotics used were not consistent across studies. A mixture of lactobacilli and bifidobacteria was used in four studies, *Lactobacillus rhamnosus HN001* and *Bifidobacterium animalis* subsp. *lactis HN019* alone were evaluated in three studies, and ten studies looked into different strains of lactobacilli. A sub-meta-analysis showed that probiotic mixtures were superior to monotherapy with either lactobacilli or bifidobacteria (RR 0.54; 95% CI: 0.43–0.68; *p* < 0.00001) in the prevention of eczema.

The second systematic review by Cuello-Garcia et al. [9] evaluated randomized-controlled trials that examined probiotic supplementation in pregnant women, breastfeeding mothers, infants, and children. The authors identified 29 randomized-controlled trials that met their inclusion criteria. Of the 29 studies, 15 trials (n = 3509) evaluated probiotic use during pregnancy. Exploration of the data showed a reduced risk of eczema in infants (RR 0.72; 95% CI: 0.61–0.85) with probiotic use during pregnancy. Thirteen studies (n = 1595) evaluating the use of probiotics in breastfeeding mothers also revealed a reduced risk of eczema (RR 0.61; 95% CI: 0.50–0.74). In addition, fifteen trials (n = 3447) evaluating the use of probiotics in infants reported a reduced risk of eczema (RR 0.81; 95% CI: 0.70–0.94).

The most recent systemic review from Li et al. [10] also assessed randomized-controlled trials of probiotics administered to infants and children in utero and/or after birth in studies published up to March 2018 and published findings similar to the two previous systematic reviews. The authors evaluated 27 randomized-controlled trials and 1 controlled cohort article that met their inclusion criteria. Analysis of these studies showed that prenatal and/or postnatal probiotic supplementation was effective in preventing atopic dermatitis in infants and children in Asia (OR 0.68; 95% CI: 0.49–0.94) and Europe (OR 0.67; 95% CI: 0.53–0.85). They also reported that the use of probiotics both during the prenatal and postnatal period reduced the incidence of atopic dermatitis (OR 0.67; 95% CI: 0.59–1.01), or only postnatal use (OR 0.66; 95% CI: 0.37–1.15) showed no statistical significance. Interestingly, receiving probiotics no more than 6 months after birth was shown to have a significantly lower incidence of atopic dermatitis (OR 0.61; 95% CI: 0.48–0.76), while administering probiotics for >12 months was not effective in preventing atopy compared to controls (OR 1.10; 95% CI: 0.80–1.51).

While the results from these three systematic reviews show some promise in the use of probiotics for the primary prevention of atopic dermatitis, more recent studies have presented different results. It is important to note that the review from Lin et al. included only one study published after 2014 (Cabana et al., 2017) [11]. This randomized double-blind placebo-controlled trial evaluated the efficacy of *L. rhamnosus GG* (LGG) supplementation in the first 6 months of life with a primary outcome of childhood eczema incidence in high-risk infants at 2 years of age and a secondary outcome of asthma incidence within 5 years of age A total of 184 infants were randomly assigned to either placebo or LGG. Participants in the probiotics arm (n = 92) received a daily dose of 10 billion colony-forming units of LGG and 225 mg of inulin. Those in the control group (n = 92) received 325 mg of inulin alone. At 2 years of age, the incidence of eczema was 30.9% (95% CI: 21.4–40.4%) in the control arm and 28.7% (95% CI: 19.4–38.0%) in the LGG arm (HR 0.95; 95% CI: 0.59–1.53; log rank *p* = 0.83). The study concluded that early supplementation with LGG in the first 6 months of life does not appear to prevent eczema at 2 years of age [11].

Second, a two-center randomized, double-blind, placebo-controlled trial evaluating maternal-only supplementation with *L. rhamnosus HN001* (HN001) did not show a significant reduction in the prevalence of eczema. Mothers participating in the study took HN001 daily from 14–16 weeks of gestation till 6 months postpartum, if breastfeeding. HN001 was not given directly to the infant. Of the 423 participants recruited, 96% (203/212) in the HN001 group and 95% (200/211) in the placebo group completed the study. The prevalence of eczema was 21.5% in the placebo group and 18% in the HN001 group (HR 0.83; 95% CI: 0.53–1.29; *p* = 0.40) [12,13]. It is important to note that Wickens et al. previously found that probiotic supplementation in both mother and infant decreased the incidence of eczema [14,15] and that their previous findings were included in the systematic reviews by Zuccotti et al., Cuello-Garcia et al., and Li et al.

While some studies show a benefit in probiotic administration to both mothers during pregnancy and infants in their first month of life, it is important to take into account the increased risk of bias and inconsistency in results along with the lack of homogeneity when evaluating probiotic strains. Further studies are needed in this area before routine recommendations can be made to patients and families.

## 3. Asthma

Atopy is a common harbinger for asthma, a chronic inflammatory condition of the airways that, when uncontrolled, can result in a poor quality of life or even death [1]. The evidence for use of probiotics as a preventative or therapeutic agent for respiratory allergies appears low. A recent mini-review by Mennini et al. highlights that in asthma, MMP9 (members of a family of enzymes that cleave extracellular matrix proteins) levels were significantly increased, and treatment with LGG was shown to decrease MMP9 expression in lung tissue and inhibit inflammatory cell infiltration [16]. Furthermore, they also highlighted that in OVA-sensitized mice, LGG suppressed the airway hyper-responsiveness to methacholine and reduced the number of infiltrating inflammatory cells and Th2 cytokines in bronchoalveolar lavage fluid and serum. LGG has previously been reported to reduce the concentration of exhaled nitric oxide among 4- to 7-year-olds in pediatric asthma [17]; however, these results could not be reproduced under similar circumstances in a later study [18].

A meta-analysis including nine different trials and a total of 3257 children showed an RR of 0.99 (95% CI: 0.81–1.21) of asthma in children receiving probiotics [19]. The majority of studies have evaluated asthma alongside eczema since they have not been adequately powered to assess the effects of probiotics on asthma alone (less prevalent than eczema in the young ages). The previously described randomized-controlled trial by Cabana et al. evaluated probiotics and the incidence of asthma at 5 years of age as a secondary outcome The study did not show a significant reduction in asthma development following the use of probiotics, with an incidence of asthma at 17.4% in the control arm (n = 92) and 9.7% in the LGG arm (n = 92) (HR 0.88; 95% CI: 0.41–1.87; log rank *p* = 0.25) [11]. These findings were also consistent with the previously mentioned systematic reviews by Zuccottti et al. and Cuello-Garcia et al., which also did not show any significant effect of probiotics on asthma [8,9]. We conclude that current evidence does not support the use of probiotics in the prevention of asthma.

## 4. Allergic Rhinitis

Allergic rhinitis affects between 10 and 30% of the population, and the management of this disease is costly, both in the amount spent on medications and in the loss of time away from work [1]. Currently, there is no strong evidence that probiotics have an effect on the development of allergic rhinitis [8], with some studies showing that there may even be an increased prevalence of allergic rhino-conjunctivitis in those who use probiotics perinatally and in infancy [20].

In a follow-up to their previous study that examined the prenatal and postnatal use of a probiotic mixture in high-risk children [18,21], Peldan et al. administered questionnaires to the parents of their study patients (at the time between 5 and 10 years of age) investigating the presence of atopy, including allergic rhinitis, in those children. Of the patients who had previously participated in the study, 407 (79.5%) from the probiotic group and 400 (79.1%) in the placebo group returned the questionnaire. Analysis of the lifetime prevalence of allergic rhinitis was the same in both placebo and probiotic group; however, the prevalence of allergic rhino-conjunctivitis at age 5–10 years was greater in the probiotic group compared to the placebo group (36.5% versus 29.0%, OR: 1.43, 95% CI: 1.06–1.94, *p* = 0.03). The authors did acknowledge the limitations of their study including the fact that a follow-up questionnaire may be subjected to bias since symptoms of viral rhinitis may be mistaken for allergic rhinitis.

## 5. Food Allergy

Food allergy describes an adverse immune response to a food allergen; it can include an immediate IgE-mediated response or a delayed cell-mediated response [22]. For the purposes of this review, food allergy pertains to the immediate IgE-mediated response. It is estimated that 240–550 million people in the world may suffer from food allergy with the prevalence increasing [1]. There is a paucity of well-designed studies examining probiotics with a primary end point of preventing food allergies or inducing tolerance.

In 2016, a meta-analysis of literature published up to July 2015 performed by Zhang et al. reported that a combined prenatal and postnatal probiotic administration reduced the risk of food sensitization (RR 0.77, 95% CI: 0.61–0.98). Data were derived from 17 different trials, and a pooled analysis revealed that only a combined approach (maternal supplementation in pregnancy and infant supplementation after birth) showed benefit. There was no effect on the risk of sensitization with prenatal or postnatal probiotic administration alone [23]. A significant limitation in studies examining the effect of probiotics in food allergy is the lack of an objective evaluation in the form of a food challenge, the gold standard for diagnosing a food allergy.

In children with peanut allergy, a study evaluated the effects of probiotics as an adjuvant to peanut oral immunotherapy (POIT). This double-blind placebo-controlled randomized trial enrolled children with confirmed peanut allergy. The 31 children allocated to the probiotic and peanut OIT (PPOIT) group received 2 × 10^10^ colony-forming units of *L. rhamnosus* CGMCC1.3724 along with peanut OIT once daily. Additionally, 31 children received both a probiotic placebo (maltodextrin) along with an OIT placebo (maltodextrin with brown food coloring and peanut essence) once daily. After receiving a peanut maintenance dose of 2 g of peanut protein for a minimum of 6 months, patients underwent a double-blind placebo-controlled (DBPC) peanut challenge. Children who were successfully desensitized subsequently underwent a second DBPC challenge to peanut following 2 or more weeks of treatment discontinuation (for the assessment of sustained unresponsiveness). At the end of the trial, the investigators reported that 82.1% in the PPOIT group achieved a sustained unresponsiveness compared to the 3.6% in the placebo group (*p* < 0.001). They also noted that 89.7% of those receiving PPOIT were desensitized compared with the 7.1% who received placebo [24]. A follow-up study 4 years after treatment cessation reported that participants from the PPOIT group were significantly more likely than those from the placebo group to have continued eating peanuts (16 (67%) of 24 versus 1 (4%) of 24; absolute difference 63% (95% CI: 42–83), *p* = 0·001). It was also noted that PPOIT-treated participants had smaller wheals in peanut skin prick test and significantly higher peanut sIgG4:sIgE ratios than placebo participants. In addition, 7 (58%) of 12 participants from the PPOIT group attained an 8-week sustained unresponsiveness, compared with 1 (7%) of 15 participants from the placebo group (absolute difference 52% (95% CI: 21–82), *p* = 0·012). The investigators concluded that PPOIT provided long-lasting clinical benefit and persistent suppression of the allergic immune response to peanut [25]. While the results of both studies suggested that probiotics may help patients achieve peanut tolerance, a significant limitation of the trial was the fact that it did not include children that received peanut OIT or probiotic as a single intervention, and it would be of great benefit to see the results of a study comparing these groups and including them into the final analysis.

In children with cow’s milk allergy, a different randomized-controlled trial evaluated the use of extensively hydrolyzed casein formula (EHCF) with and without *L. rhamnosus GG* (LGG). A total of 220 children with confirmed cow’s milk allergy were randomized to either receive EHCF alone or EHCF with LGG. The primary outcome was any allergic manifestation including eczema, asthma, or allergic rhino-conjunctivitis during the 36 months of the study. The authors found that the use of EHCF with LGG decreased the incidence of allergic manifestations including asthma, eczema, and allergic rhino-conjunctivitis over a 3-year period with a number of children needed to be treated being 4 (absolute risk difference −0.23; 95% CI: −0.36 to −0.10; *p* < 0.001). They also reported that the arm receiving EHCF with LGG favored the development of oral tolerance in children with IgE-mediated cow’s milk allergy at 36 months (absolute risk difference 0.27; 95% CI: 0.11–0.43; *p* < 0.001) [26]. While the family pediatricians in the study were blinded to which patients were receiving LGG, it was important to note that a significant limitation to this trial was that the parents were not blinded to which formula their child was receiving.

Evidence at this time for probiotics, either as a preventative or therapeutic agent for food allergies remains low, and probiotics are currently not recommended for routine use in food allergy prevention.

## 6. Safety of Probiotics

Probiotics are generally considered safe; however, they may not be completely innocuous. While there are some probiotic products that are considered medicinal, most are classified as commercial food/dietary supplements or natural health products. As a result, the probiotics that are readily available to the general public are not subjected to the same oversight as their medicinal counterparts putting people at risk for contamination of other products (i.e., cow’s milk in those with cow’s milk allergy) and mislabeling of the potency and species of probiotics [27]. The authors rightfully recommend that patients should always read labels closely and use commercial probiotics with caution.

Probiotics have also been reported to cause endocarditis in those with structural heart defects [28,29,30] and promoting d-lactic acidosis resulting in encephalopathy [31] and decreased mental acuity [32], although this remains controversial and needs to be further explored [33]. In a review by Doron and Syndman examining the adverse events associated with probiotics, at least eight cases of bacteremia were associated with lactobacilli, nine cases of sepsis with *Saccharomyces boulardii*, *Lactobacillus rhamnosus GG*, *Bacillus subtilis*, *Bifidobacterium breve*, or combination probiotics, and two cases of endocarditis were due to lactobacillus and streptococcus probiotics [34]. Some of these cases occurred in the setting of intensive care units, where patients were likely to be immunocompromised and central venous catheters were in place, thus increasing the risk of infection [34,35].

Perhaps the most cited study pertaining to adverse effects of probiotic use comes from the Dutch acute pancreatitis study group [36]. In 2008, the group conducted a randomized double-blind placebo-controlled trial examining the use of probiotics in the setting of acute pancreatitis. The study randomly assigned 298 patients diagnosed with severe acute pancreatitis to receive enterally a mixture of probiotics (*Lactobacillus acidophilus*, *Lactobacillus casei*, *Lactobacillus salivarius*, *Lactococcus lactis*, *Bifidobacterium bifidum*, *Bifidobacterium lactis*) twice a day or placebo. Treatment was started within 72 h of symptom onset and was continued for 28 days. Of the 298 patients enrolled, 153 were assigned to receive probiotics and 145 received placebo. The primary endpoint was the combination of any infectious complications including infected pancreatic necrosis, bacteremia, pneumonia, urosepsis, or infected ascites either during admission or at 90-day follow-up. Both groups were equivocal in demographics, clinical characteristics, severity of symptoms, and baseline laboratory findings. There was no significant difference in the primary endpoint of infectious complications with 46 (30%) in the probiotics arm and 41 (28%) in the placebo arm (RR 1.01, CI: 95% 0.75–1.51). However, difference in mortality was significant with 24 (16%) in the probiotic group versus 9 (6%) in the placebo group (RR 2.53, CI: 95% 1.22–5.25), with 9 cases from the probiotic cohort dying from bowel ischemia, which the authors speculated might have been due to local infection from probiotics as well as increased oxygen demand in those areas. The authors concluded that probiotics should not be administered in critically ill patients or those at risk for non-occlusive bowel ischemia.

A more recent systematic review examining the efficacy and safety of probiotics in people with cancer included 25 studies (n = 2242 participants) in their safety analysis [37]. Randomized-controlled trials, non-randomized studies, and case reports of patients diagnosed with cancer who received probiotics as an intervention, were included. Five case reports were identified involving probiotic-associated infections including bacteremia and fungemia. Two deaths were reported in patients receiving probiotics; however, these were not attributed to the probiotic intervention. One cohort study specifically reported the absence of probiotic-associated infections. The authors of the review could not reach a conclusion with confidence in regards to the safety of probiotics as there were inconsistencies in the reporting and defining of adverse events in different studies as well as noticeable heterogeneities including the strains of probiotics used, duration of treatment, and dosage.

While reports of sepsis due to lactobacillus or bifidobacterium exist, these species are generally not considered pathogenic. Relevant case reports are usually associated with an underlying comorbidity resulting in immunocompromise [38]. Overall, the risk with probiotic administration appears low in non-immunocompromised patients, but good clinical judgment is still important in making the decision of whether to administer probiotics or not in different clinical settings.

## 7. Conclusions

Current guidelines from prominent medical societies including the American Academy of Pediatrics [39], the European Academy of Allergy and Clinical Immunology [40,41], the National Institute of Allergy and Infectious Disease [42], and the European Society for Pediatric Gastroenterology, Hepatology, and Nutrition [43] do not recommend the use of probiotics for primary prevention of allergic disease [44]. The World Allergy Organization (WAO) does not support the use of probiotics for primary prevention of allergic disease, but does favor probiotic supplementation in pregnant/lactating women and in infants with a family history of allergic disease [45]. These recommendations, however, are conditional recommendations from very low-quality evidence based mostly on the “unlikely to harm” principle rather than strong evidence for efficacy of the intervention. It is important to note that there are no recommendations currently for a specific strain of probiotic, as most studies show a significant heterogeneity in strains used and cannot come to a conclusion as to which strain is the most effective [45]. As a result of the previously highlighted lack of homogeneity in specific probiotic strains, many trials are now carefully recording the specific strains of probiotics that are being used, in an attempt to achieve consistency in reported data across different research studies [46,47,48,49].

In conclusion, current evidence does not support the routine use of probiotics as an intervention for preventing any form of allergic disease, with the exception of eczema in high-risk infants (WAO recommendation) [45]. The optimal strains, dosages, timing, and duration of probiotic administration remain unknown. However, research in this area is ongoing and will hopefully provide better insights into how probiotics may contribute to the prevention or treatment of atopic diseases.
